# Cross-sectional survey to determine fatigue in patients with systemic lupus erythematosus

**DOI:** 10.3389/fpsyg.2024.1376257

**Published:** 2024-07-24

**Authors:** Jingya Lu, Minmin Yu, Rong Xu

**Affiliations:** Department of Rheumatology and Immunology, The First Affiliated Hospital of Soochow University, Suzhou, China

**Keywords:** fatigue, systemic lupus erythematosus, treatment, care, clinical

## Abstract

**Background:**

Fatigue is a prevalent symptom among individuals with active systemic lupus erythematosus (SLE). We aimed to investigate the status quo and influencing factors of fatigue in patients with SLE, to provide insights to the clinical SLE treatment and care.

**Methods:**

We conducted a longitudinal survey on the fatigue of active SLE patients from June 2022 to November 2023 in our hospital. Fatigue severity scale (FSS), Self-rating Anxiety Scale (SAS) and Self-Rating Depression Scale (SDS) were used for data collection. Pearson correlation and multiple stepwise regression analysis were conducted to analyze the relevant factors affecting the fatigue level of patients with SLE.

**Results:**

A total of 212 active SLE patients were included, the incidence of fatigue in active SLE patients was 55.66%. There were statistical differences in the fatigue score between SLE patients with different age and course of SLE (all *p* < 0.05). Fatigue was positively correlated with anxiety (*r* = 0.559) and depression (*r* = 0.591) in patients with SLE (all *p* < 0.05). Multiple stepwise regression analysis indicated that age, course of SLE, anxiety and depression were the influencing factors of fatigue in SLE patients (all *p* < 0.05).

**Conclusion:**

Patients with SLE exhibit a significant degree of fatigue, which varies with respect to age, disease duration, and the presence of comorbid anxiety and depression. It is imperative that healthcare providers closely monitor the fatigue levels in this patient population and implement targeted interventions to mitigate the impact of fatigue on the quality of life and overall well-being of individuals with SLE.

## 1 Background

Systemic lupus erythematosus (SLE) is a systemic autoimmune disease with clinical manifestations of systemic multi-system and multi-organ damage (Zucchi et al., 2022). SLE has a long course and repeated episodes of flares and remission (Lazar and Kahlenberg, 2023; Zucchi et al., 2023). SLE is more common in young women, and the age of the disease is mainly 20 ~ 40 years old (Crow, 2023). The incidence of SLE in China is about 1/1000 (Jiang et al., 2021). At present, the pathogenesis of SLE is not completely clear, which may be caused by genetic, environmental, infection and other factors (Fasano et al., 2023). Active SLE patients are often complicated with fatigue, infection and other adverse symptoms due to the disorder of autoimmune function and the extensive use of hormones and immunosuppressants (Caielli et al., 2023; Frostegard, 2023). Therefore, a thorough understanding of the epidemiological traits and determinants of adverse symptoms among patients with active systemic lupus erythematosus (SLE) is essential. Such insights are pivotal for informing the development of evidence-based clinical nursing strategies tailored to the needs of this patient cohort.

Fatigue is one of the most common clinical manifestations of SLE (Kaul et al., 2016). Fatigue is a subjective feeling of tiredness or burnout, which may be related to activity (Krupp et al., 1989). Fatigue has been reported to exert a significant impact on the quality of life in patients, potentially correlating with diminished cognitive performance, increased incidence of behavioral and cognitive errors, as well as the emergence of negative affective states such as anxiety and depression. (Dukes et al., 2021; Hosey et al., 2021; Maisel et al., 2021). Therefore, the objective of the present study is to delineate the prevalence and determinants of fatigue among individuals with SLE. This investigation aims to generate empirical data that can inform the development of targeted fatigue management protocols and enhance the provision of specialized nursing care for SLE patients.

## 2 Methods

### 2.1 Ethics

This study was a cross-sectional survey, the study had obtained the ethical approval from the ethics committee of our hospital (approval number: 21058). And all the patients were well informed and signed the written informed consents.

From June 2022 to November 2023, we conducted a longitudinal survey of active SLE patients in the Department of Rheumatic Immunology at the First Affiliated Hospital of Soochow University. The inclusion conditions of the study population in this study were as follows: the diagnosis of SLE conformed to the SLE classification criteria revised by the American Rheumatic Association; those with systemic lupus erythematosus disease assessment index (SLEDAI) score ≥ 9, who were active SLE patients; those with normal reading and expression skills; and those volunteered to participate in this survey. The exclusion criteria of this study were as follows: patients with disturbance of consciousness and communication; patients who refused to participate in this survey.

### 2.2 Survey tool

#### 2.2.1 General information questionnaire

We have designed a general information questionnaire to collect some relevant data about patients, including gender, age, marital status, education level, medical expenses payment method, home place, family monthly income and course of SLE.

#### 2.2.2 Fatigue severity scale

We used the FSS to measure the fatigue degree of SLE patients. The FSS was a standardized and verified scale (Schwartz et al., 1993), which mainly evaluated the degree of physical and mental fatigue of patients, including 9 items, with items ranging from strong opposition (1 point) to strong agreement (7 points). The total FSS score ranged from 9 to 63 points. The FSS score ≥ 36 indicated that the patient had fatigue. Previous studies (Mao, 2015; Chen and Wu, 2016) had shown that the Cronbach’s α coefficient of the scale is 0.63 ~ 0.86 and the test–retest reliability is 0.55 ~ 0.77, indicating that FSS had good reliability and validity.

#### 2.2.3 Self-rating anxiety scale

SAS, developed by the scholar William W. K. Zung in 1971, serves as an instrument for the self-assessment of an individual’s anxiety levels (Dunstan and Scott, 2020; Shao et al., 2020). SAS had a total of 20 items, each item using the Likert 4 rating scale, from “no or very little time” to “most or all of the time” 1–4 points. The sum of the scores of each item was multiplied by 1.25 as the standard total score, the higher the score, the more serious the anxiety. The total score 50–59 was considered as mild anxiety, 60–69 was considered as moderate anxiety, 70 was considered as severe anxiety. The scale has been widely used and has good reliability and validity (Shao et al., 2020; Yue et al., 2020).

#### 2.2.4 Self-rating depression scale

SDS was developed by the scholar Zung in 1965 and is a tool for self-assessment of depression (Zung, 1965). A total of 20 items, using the Likert 4-grade scoring method, the positive items scored 1to 4 points. The severity of depression is usually expressed by severity index. The severity of depression was usually expressed by severity index. Depression severity index = cumulative score of each item / 80, <0.50 was no depression, 0.50–0.59 was mild depression, 0.60–0.69 was moderate depression, ≥0.70 was severe depression. SDS has demonstrated robust psychometric properties, including good reliability and validity, and has been extensively utilized in various research contexts (Jokelainen et al., 2019).

### 2.3 Survey investigation

All the questionnaires were answered anonymously, and the researched explained to the respondents the purpose of the survey, the method of filling in the questionnaire, matters needing attention, the anonymity and confidentiality of the commitment data, and the respondents completed the questionnaire independently. We collected the questionnaire on the spot. After all the questionnaires were collected, the researchers themselves checked the quality of the questionnaires to ensure the validity of the questionnaires.

### 2.4 Statistical method

In this study, SPSS 23.0 software was used for statistical analysis, the counting data were described by frequency and constituent ratio, and the measurement data were described by mean ± standard deviation. We used t-test, one-way ANOVA, Pearson correlation analysis and multiple stepwise regression analysis to analyze the relevant factors affecting the fatigue level of patients with SLE.

The test level of the statistical value of this survey was α = 0.05.

## 3 Results

**Figure 1:**
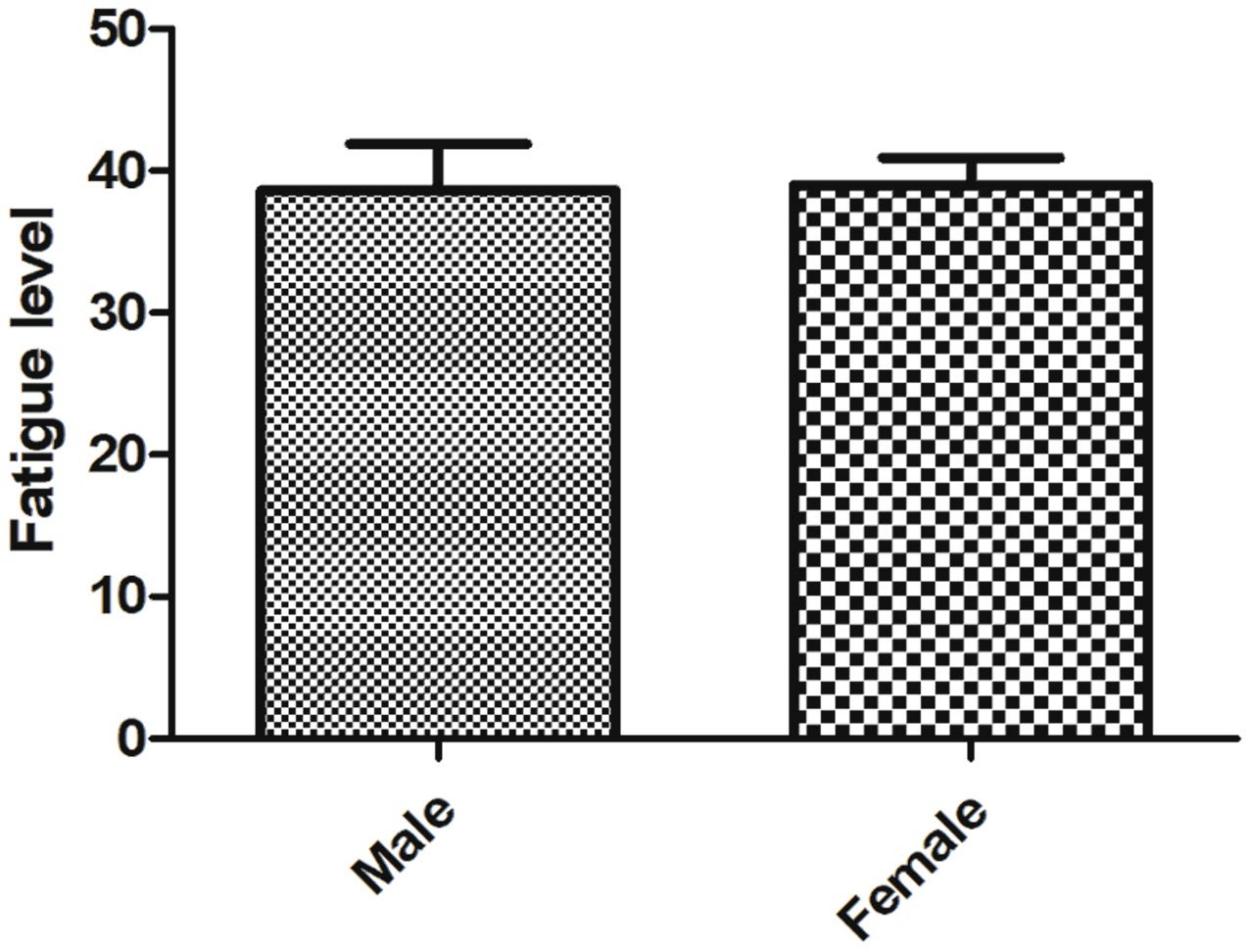
The fatigue score of male and female patients with SLE.

A total of 212 active SLE patients were included. The FSS score of 118 patients had ≥36, indicating that they had certain degree of fatigue, the incidence of fatigue in active SLE patients was 55.66%. As shown in Table 1, there were statistical differences in the fatigue score between SLE patients with different age and course of SLE (all *p* < 0.05). There were no statistical differences in the fatigue score between SLE patients with different gender (Figure 1), marital status, educational level, medical expenses payment method, home place and family monthly income (all *p* > 0.05).

**Table 1:** The characteristics of included SLE patients (*n* = 212).

Characteristics	Cases (*n* = 212)	Fatigue score	t/F	P
Gender			2.154	0.074
Male	78 (%)	37.10 ± 6.14		
Female	134 (%)	37.51 ± 4.97		
Age(years)			1.089	0.016
<30	50	35.80 ± 5.33		
30 ~ 50	138	38.85 ± 4.59		
>50	24	37.01 ± 6.12		
Marital status			1.837	0.113
Married	145 (%)	37.81 ± 7.45		
Unmarried	44 (%)	37.28 ± 6.40		
Divorced	23 (%)	38.72 ± 6.03		
Education level			1.202	0.086
Primary school	12 (%)	37.07 ± 4.28		
Junior high school	46 (%)	36.14 ± 6.22		
Senior middle school	61 (%)	37.85 ± 5.35		
University	93 (%)	37.27 ± 5.88		
Medical expenses payment method			2.052	0.167
Self-paid	70 (%)	38.93 ± 6.19		
Medical insurance	142 (%)	37.61 ± 7.23		
Home place			2.131	0.084
City	138 (%)	37.48 ± 5.42		
Rural area	74 (%)	37.50 ± 6.29		
Family monthly income(RMB)			1.286	0.151
<3,000	26 (12.26%)	37.83 ± 6.78		
3,000 ~ 6,000	138 (65.09%)	37.42 ± 6.18		
>6,000	48 (22.64%)	37.58 ± 5.44		
Course of SLE			2.175	0.029
<3y	145 (68.40%)	37.42 ± 6.07		
≥3y	67 (31.60%)	38.25 ± 5.61		

**Figure 2:**
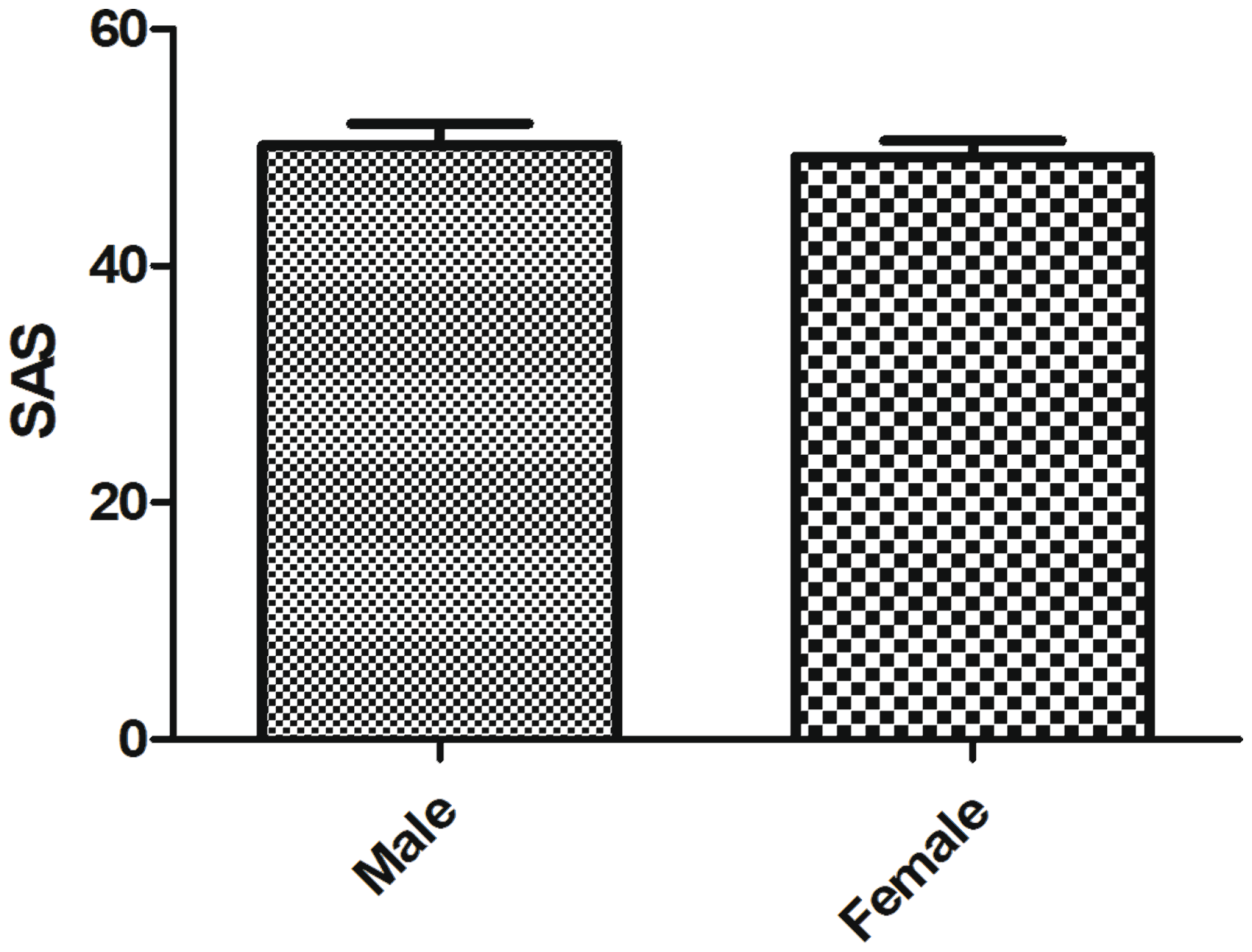
The SAS score of male and female patients with SLE.

As shown in Table 2, the average SAS score of SLE patients was 50.71 ± 7.85, including no anxiety in 101cases, mild anxiety in 79 cases, moderate anxiety in 22 cases and severe anxiety in 10 cases. No statistical difference in the SAS score was observed between male and female SLE patients (Figure 2).

**Table 2:** The anxiety level of SLE patients.

	Cases
No anxiety (SAS <50)	101 (47.64%)
Mild anxiety (SAS 50 ~ 59)	79 (37.26%)
Moderate anxiety (SAS 60 ~ 69)	22 (10.38%)
Severe anxiety (SAS ≥ 70)	10 (4.72%)

SAS, Self-rating anxiety scale.

**Figure 3:**
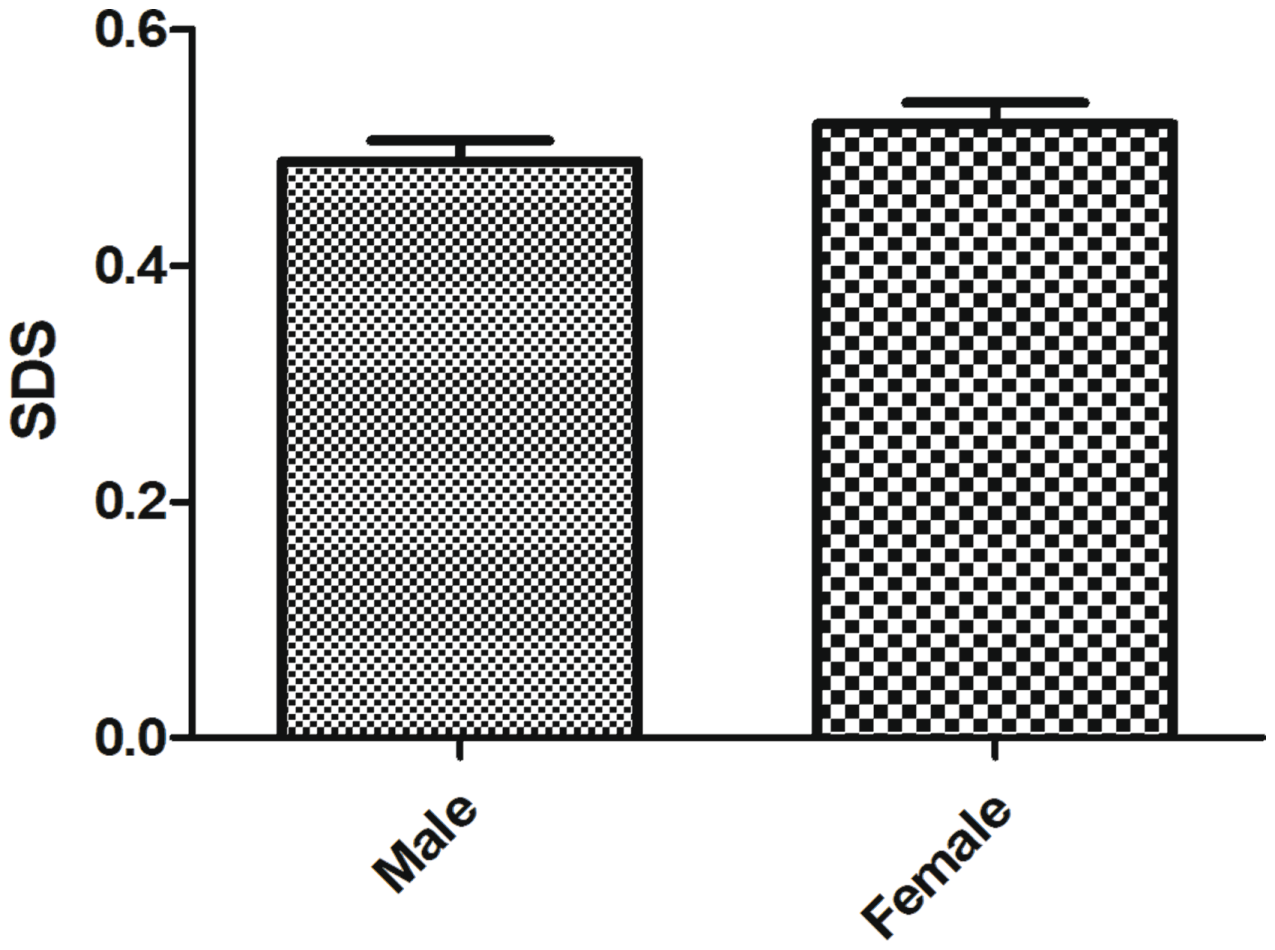
The SDS score of male and female patients with SLE.

As shown in Table 3, the average score of depression severity index in SLE patients was 0.51 ± 0.23. There was no depression in 134 cases, 63 mild depression cases, 12 moderate depression cases and 3 severe depression cases. No statistical difference in the SDS score was observed between male and female SLE patients (Figure 3).

**Table 3:** The depression level of SLE patients.

	Cases
No depression (SDS <0.5)	134 (63.21%)
Mild depression (SDS 0.5 ~ 0.59)	63 (29.72%)
Moderate depression (SDS 0.6 ~ 0.69)	12 (5.66%)
Severe depression (SDS ≥ 0.7)	3 (1.42%)

SAS, Self-rating depression scale.

**Figure 4:**
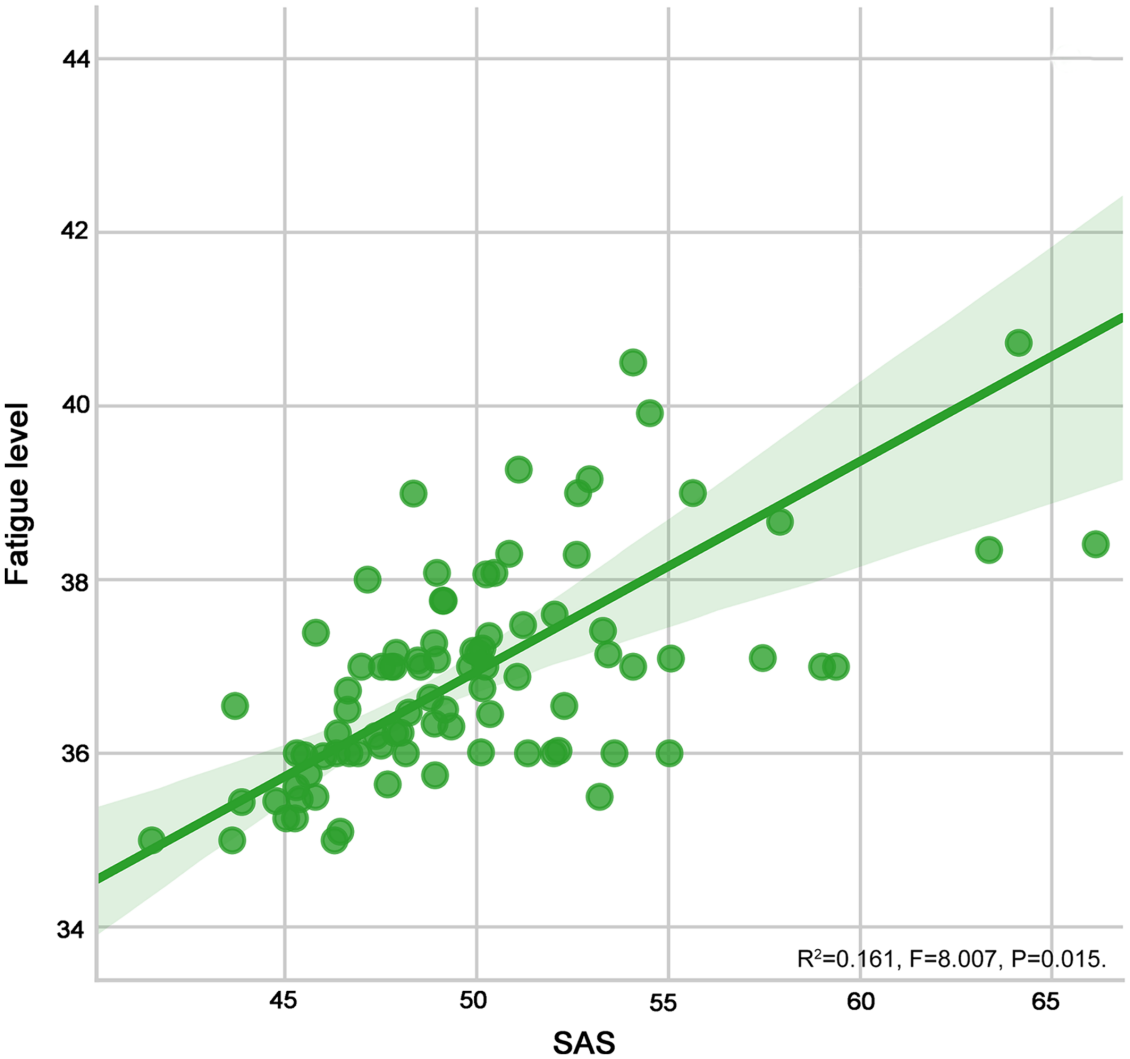
The scatter graph for the relation of fatigue versus anxiety.

**Figure 5:**
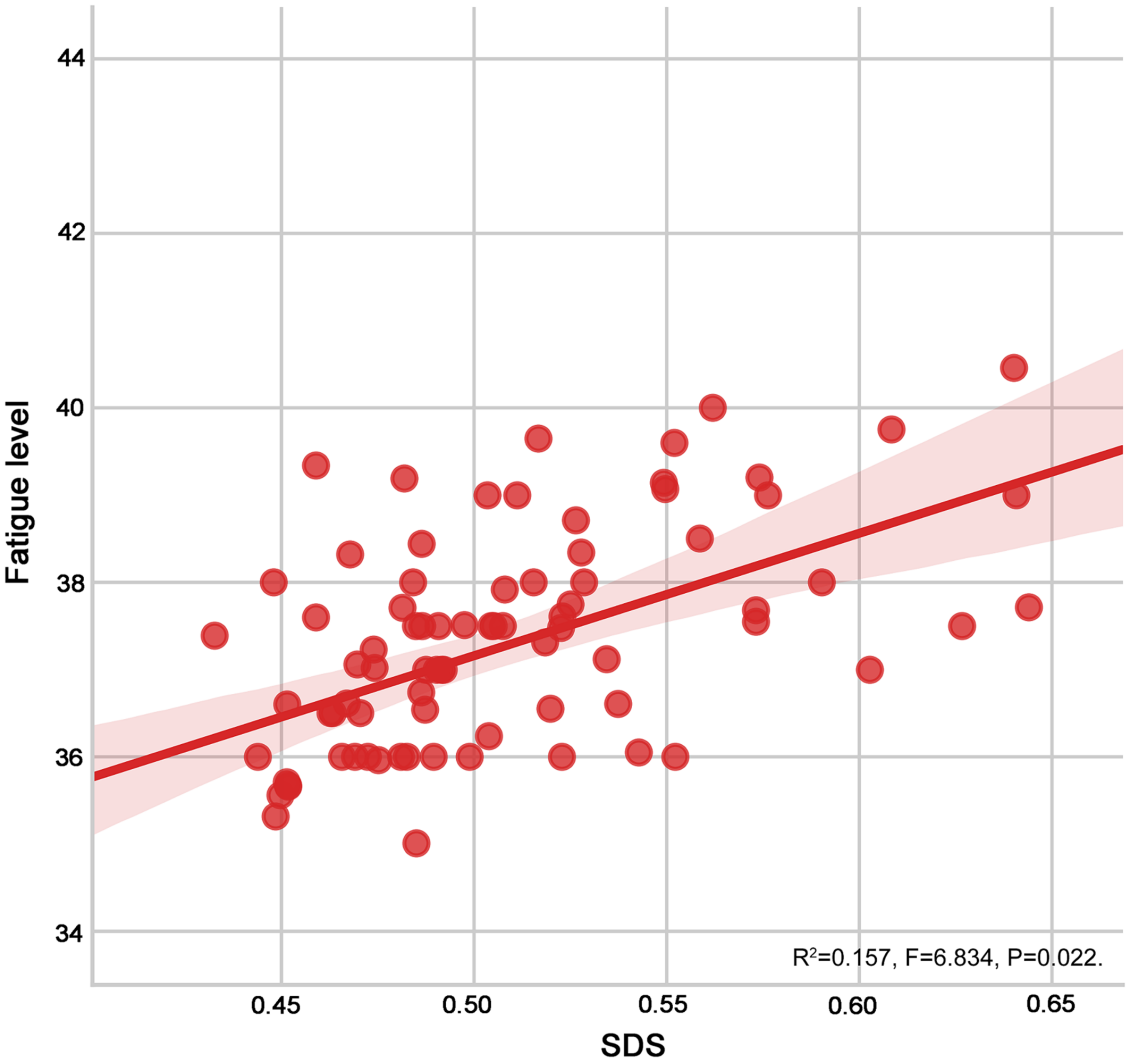
The scatter graph for the relation of fatigue versus depression.

As shown in Table 4 and Figures 4, 5, fatigue was positively correlated with anxiety (*r* = 0.559) and depression (*r* = 0.591) in patients with SLE (all *p* < 0.05).

**Table 4:** The correlation of fatigue and anxiety and depression of SLE patients.

Item	Fatigue score	*P*
Anxiety	r = 0.559	0.013
Depression	r = 0.591	0.007

As indicated in Table 5, multiple stepwise regression analysis indicated that age, course of SLE, anxiety and depression were the influencing factors of fatigue in SLE patients (all *p* < 0.05).

**Table 5:** Multiple stepwise regression analysis on the influencing factors of fatigue in SLE patients.

						95%CI	
Items	*B*	*E*	*β*	*t*	*P*	Lower limit	Upper limit
Constant	56.05	4.85		12.81	<0.001	45.93	60.20
Age	2.25	0.24	−0.35	−2.48	0.016	1.21	3.56
Course of SLE	1.28	0.41	0.22	3.75	<0.001	0.88	1.95
Anxiety	2.05	1.95	−0.45	−3.11	<0.001	1.23	3.01
Depression	1.74	1.03	0.12	2.26	0.018	0.83	2.35

Adjusted *R*^2^ = 0.497.

## 4 Discussions

SLE is an autoimmune inflammatory connective tissue disease involving multiple organs (Bakshi et al., 2018). Previous studies (Mertz et al., 2020; Aranow et al., 2021) have shown that fatigue is the most common symptom in patients with SLE, and its incidence can be as high as 80%. Many studies (Ramsey-Goldman and Rothrock, 2010; Mahieu and Ramsey-Goldman, 2017) have shown that the disease associated fatigue is affected by many factors, such as long-term treatment, fear and worry about the prognosis, pain and so on. It has been reported that although the disease symptoms of inactive SLE patients have been alleviated, but because the disease cannot be cured, long-term medication, the disease may occur repeatedly, SLE patients are under multiple physical and psychological pressure, so they are more likely to have fatigue than ordinary people (Balsamo and Santos-Neto, 2011). The results of this study have indicated that the incidence of fatigue in active SLE patients is 55.66%, and age, course of SLE, anxiety and depression are the influencing factors of fatigue in SLE patients, targeted countermeasures and nursing care are needed to reduce the fatigue in SLE patients.

The fatigue degree of active SLE patients aged 30–50 years is higher than that of other age groups. SLE patients in this age group are in middle age and bear many responsibility (Cleanthous et al., 2012). Patients not only have to face the pressure of disease, but also bear the pressure from work and family (Tarazi et al., 2019). From the perspective of physiological factors, because SLE often occurs in women, the hormone secretion of female patients of this age begins to change and gradually enter menopause, and some common menopausal symptoms such as fear and anxiety begin to appear (Monahan, 2021; Pinto et al., 2021). Under the influence of dual factors, patients often feel physically and mentally exhausted (del Pino-Sedeno et al., 2016). Health professionals should pay attention to the assessment of the psychological needs of patients of this age, strengthen psychological counseling, encourage patients to express their needs and inner feelings, adjust their mentality in time, and guide patients to engage in the activities they are interested in, so as to maintain an optimistic mood and face SLE in a positive way (Jones et al., 2014; Zhang et al., 2022).

Among patients with active SLE, SLE patients with a course of more than 3 years had a significantly higher degree of fatigue, which may be related to the longer duration of illness in patients with a longer course of disease. Previous studies (Li et al., 2016; Wan et al., 2022) have shown that there is a significant statistical significance between the score of self-rating symptom scale and the course of disease in patients with SLE. Patients with a long course of disease have more times of disease recurrence, and the organ damage is more serious, which has a negative impact on psychology (Li et al., 2019; Xue et al., 2020). The disease condition can make patients have a strong sense of out of control, which affects their ability to perceive and adjust their emotional state and confidence (Feng et al., 2019).

Our findings underscore the necessity for healthcare professionals to facilitate a rational approach to the illness among patients, bolstering their therapeutic and life confidence. Concurrently, it is imperative to foster the development of robust social support networks and to promote the adoption of constructive coping mechanisms (Wei et al., 2021). Healthcare professionals are encouraged to enhance communication with both patients and their families, thereby enabling the family unit to offer efficacious emotional and material support throughout the patient’s rehabilitation process (Andayani et al., 2022). Concurrently, healthcare professionals are advised to implement dynamic and efficacious educational models for rehabilitation, such as peer-to-peer mentorship, to augment patients’ capacity for self-management of SLE, thereby potentially mitigating and retarding the progression of the disease.

Anxiety is the main influencing factor of fatigue in SLE patients, and there is a positive correlation between anxiety and fatigue, indicating that the more serious the anxiety, the more serious the fatigue of SLE patients. SLE patients have a strong sense of uncertainty about the prognosis of the disease, coupled with the active SLE patients need to limit contact with the population, the physical and social functions of patients are damaged, resulting in anxiety (Arnaud et al., 2021). The heavy economic burden caused by the continuous medical expenses is also an important reason for the anxiety of patients. Anxiety is a psychological stress factor, which makes the body’s defense mechanism more sensitive and makes it in a state of long-term preparation (Zhang et al., 2017). When stimulated, it will lead to increased inflammatory reaction and aggravate fatigue of patients with symptoms such as nausea, anorexia and drowsiness. In addition, the higher the level of anxiety, the more sensitive the physiological reaction, which increases the burden of subjective symptoms such as pain and nausea, resulting in increased fatigue, reduced treatment compliance and prolonged hospitalization (Meszaros et al., 2012; Tisseverasinghe et al., 2018). Therefore, medical staff should pay attention to the anxiety of SLE patients, explain the knowledge related to SLE disease to patients, and teach patients how to reduce anxiety, so as to reduce the fatigue of SLE patients.

There is a positive correlation between depression and fatigue, that is, the more serious the depression, the more serious the fatigue. The possible reason is that patients with depression tend to have symptoms such as sleep disorders, loss of appetite, delayed thinking, etc. (Ji et al., 2012; Yamin et al., 2022). It will also make patients lose faith in treatment, face everything with negative coping styles, and hinder the smooth progress of treatment and communication between doctors and patient (Kelley and Kelley, 2014). Depression may have a greater impact on the prognosis of patients than heart disease, diabetes, cerebrovascular disease (MA et al., 2022). Therefore, early identification of depression is of great significance to improve the fatigue of SLE patients. Clinical medical staff should pay attention to the psychological changes of patients as soon as possible, do a good job of depression screening and long-term monitoring, and give psychological guidance to SLE patients with depression and pay attention to the occurrence of fatigue.

## 5 Conclusion

In conclusion, the findings from this survey underscore that the prevalence of fatigue among patients with SLE is 55.66%. Age, disease duration, and the presence of comorbid anxiety and depression have been identified as the principal determinants of fatigue in this patient population. Given the high incidence of fatigue in SLE patients, healthcare professionals are encouraged to implement early detection strategies and evidence-based nursing interventions that address these influencing factors, thereby potentially diminishing the incidence and severity of fatigue in SLE patients.

## Data availability statement

The original contributions presented in the study are included in the article/supplementary material, further inquiries can be directed to the corresponding authors.

## Ethics statement

The studies involving humans were approved by the study has been reviewed and approved by the ethics committee of the First Affiliated Hospital of Suzhou University (approval no. 21058). Written informed consents had been obtained from all the included patients. The studies were conducted in accordance with the local legislation and institutional requirements. The participants provided their written informed consent to participate in this study.

## Author contributions

JL: Writing – original draft, Investigation. MY: Writing – original draft, Investigation. RX: Writing – original draft, Investigation.
